# Cricotracheostomy for Anatomically Challenging Severe Scoliosis: A Report of Two Adult Cases

**DOI:** 10.1155/crot/6355617

**Published:** 2025-12-25

**Authors:** Satoko Kubo, Takashi Anzai, Shinichi Ohba, Akihisa Yoshikawa, Yusuke Takata, Masahiro Nakamura, Kumiko Tanaka, Mari Kameda, Fumihiko Matsumoto

**Affiliations:** ^1^ Department of Otorhinolaryngology, Faculty of Medicine, Juntendo University, Tokyo, Japan, juntendo.ac.jp

**Keywords:** cricotracheostomy, scoliosis, tracheostomy

## Abstract

Cricotracheostomy is a modified tracheostomy technique that involves partial resection of the anterior cricoid cartilage, which enables high‐level airway access while minimizing the risk of subglottic stenosis. This report describes two adult cases of congenital scoliosis with complex skeletal deformities, including tracheal deviation and restricted neck extension, wherein conventional tracheostomy was challenging. In both cases, cricotracheostomy successfully provided stable airway access without complications such as granulation tissue formation, infection, or subglottic stenosis during follow‐up. This report supports the utility of cricotracheostomy as the primary surgical approach in patients with severe skeletal deformities.

## 1. Introduction

Tracheostomy is a standard surgical procedure for achieving airway access, but it can be technically demanding in patients with anatomical complexities, such as a high body mass index (BMI), limited cervical extension, skeletal deformities, or aberrant vascular anatomy. This can also be particularly challenging among patients with severe scoliosis due to the presence of tracheal deviation, thoracic deformity, and difficulties in achieving proper positioning for surgery. In such cases, airway access is not only technically difficult but also psychologically stressful for the operating surgeon.

Cricotracheostomy, first introduced by Kano et al. in 2017, is a modified tracheostomy technique that involves partial resection of the anterior cricoid cartilage, enabling high‐level tracheostomy while minimizing the risk of subglottic stenosis [1]. Its utility has been demonstrated in cases involving head and neck tumors [1], aberrant carotid anatomy [2], and severe kyphoscoliosis [3, 4]. Additionally, Nanjo et al. reported its safety and feasibility in elderly, obese, and anatomically complex populations, with a low incidence of complications and reliable stoma closure [5].

This case report describes two adults with congenital severe scoliosis who presented with significant anatomical airway challenges, wherein cricotracheostomy was successfully employed as the primary surgical approach.

## 2. Case Presentation

### 2.1. Case 1

A 29‐year‐old male with a chromosomal abnormality (46, XY, −21 + der(21) + t(13; 21)(q12.3; p12)) and severe scoliosis required endotracheal intubation for respiratory support (Figures 1(a) and 1(b)). He was 150‐cm tall and weighed 29 kg (BMI: 21.5). Tracheostomy was planned for long‐term airway management in this patient, after unsuccessful attempts at ventilator weaning. Although there was a sufficient distance between the cricoid cartilage and the clavicle (about 30 mm in CT images), the patient’s neck was fixed in a rightward rotated and extended position, resulting in the trachea running obliquely from the upper right to the lower left (Figures 1(c) and 1(d)). In addition, the patient was unable to assume a fully supine position. These conditions made safe tracheostomy technically difficult. Accordingly, cricotracheostomy was selected because it does not require manipulation of the thyroid gland, carries a lower risk of intraoperative bleeding, and allows creation of a high‐level tracheal stoma, which may help prevent postoperative complications such as inappropriate tracheal tube pressure and tracheoinnominate artery fistula associated with tracheal deviation and severe thoracic curvature.

Figure 1Physical findings and radiological examinations in Case 1. (a‐b) Photographs demonstrating the severe thoracic deformity and shortened neck of the patient. The white horizontal line indicates the anatomical horizontal plane. (c) Computed tomography image demonstrating rightward displacement of the trachea due to spinal curvature. (d) Preoperative chest radiograph marked scoliosis from the cervical to the thoracic spine. (e) Postoperative chest radiograph showing the tracheostomy tube was appropriately positioned at a high level.

**Figure:**
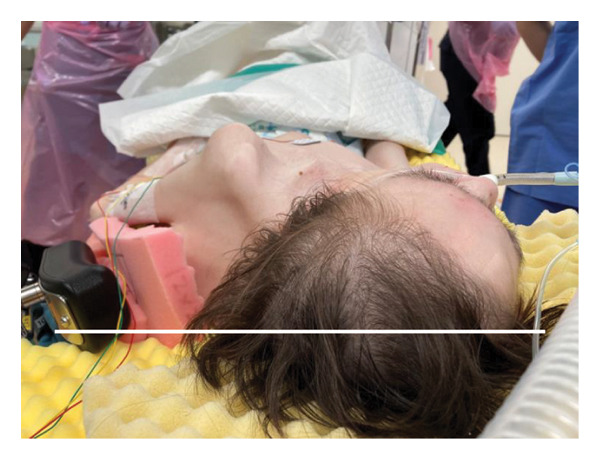
(a)

**Figure:**
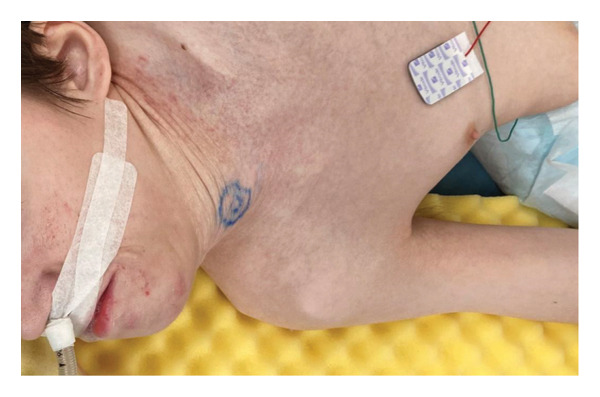
(b)

**Figure:**
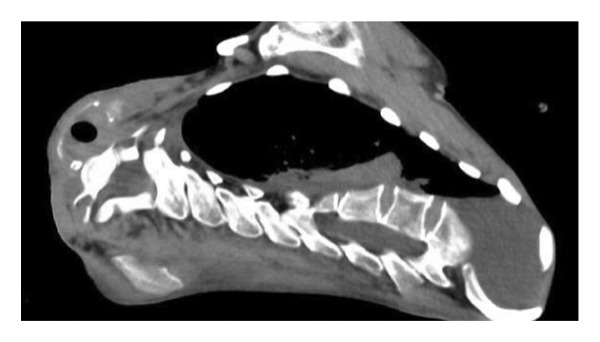
(c)

**Figure:**
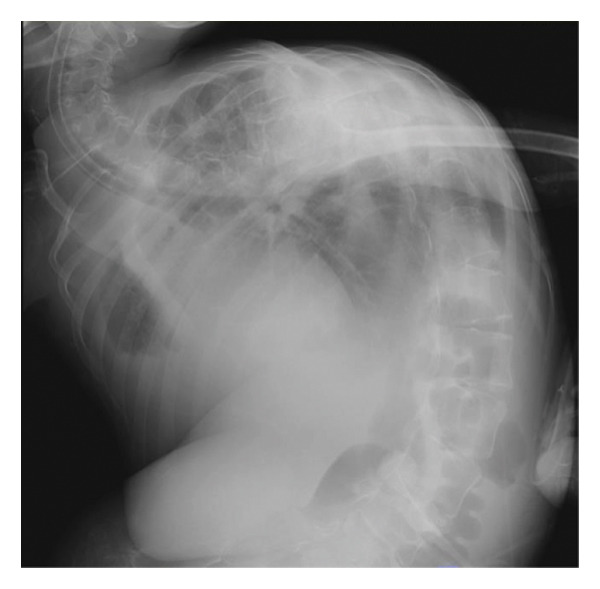
(d)

**Figure:**
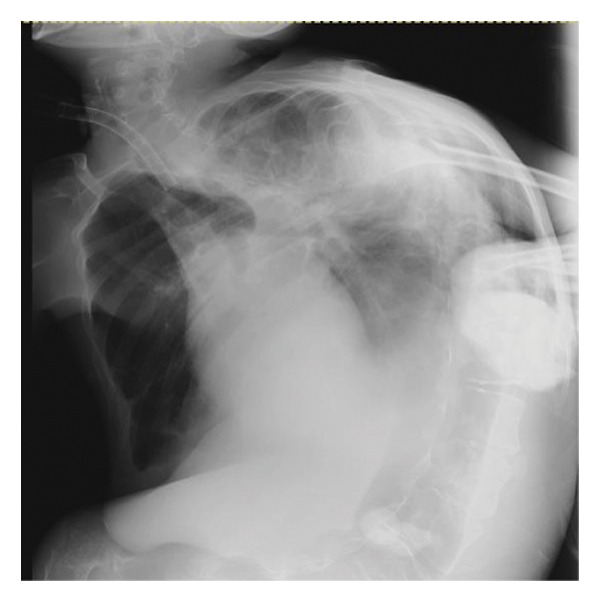
(e)

During surgery, the patient was placed in a neck‐extended position, and the cricoid cartilage was identifiable, serving as a reliable surgical landmark. A vertical midline incision was made just above the cricoid cartilage (Figure 2(a)), and the anterior one‐third of the cartilage was resected (Figures 2(b) and 2(c)). No anatomical abnormalities were observed intraoperatively in the cricoid cartilage, trachea, thyroid gland, or surrounding vascular structures.

Figure 2Surgical procedure for cricotracheostomy. (a) Skin incision line (purple line) marked above the cricoid cartilage. (b) Cricoid cartilage exposed after dissecting the anterior cervical muscles (white arrowhead). (c) Anterior portion of the cricoid cartilage resected. (d) The cricothyroid membrane is incised in an H‐shaped fashion, followed by caudal extension of the tracheal incision. (e) Formation of a stable tracheostoma. (f) Placement of a spiral‐reinforced tracheostomy tube with an adjustable neck flange.

**Figure:**
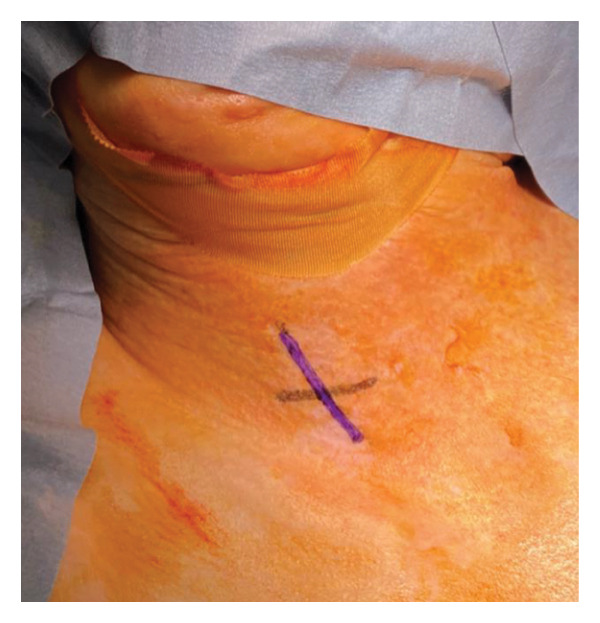
(a)

**Figure:**
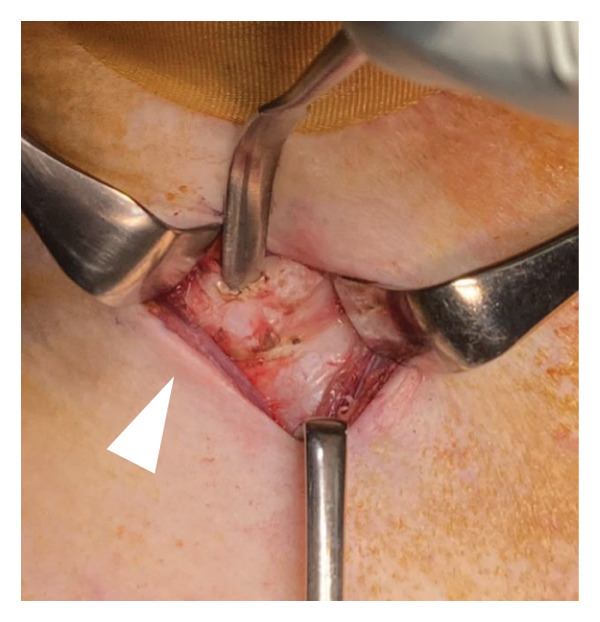
(b)

**Figure:**
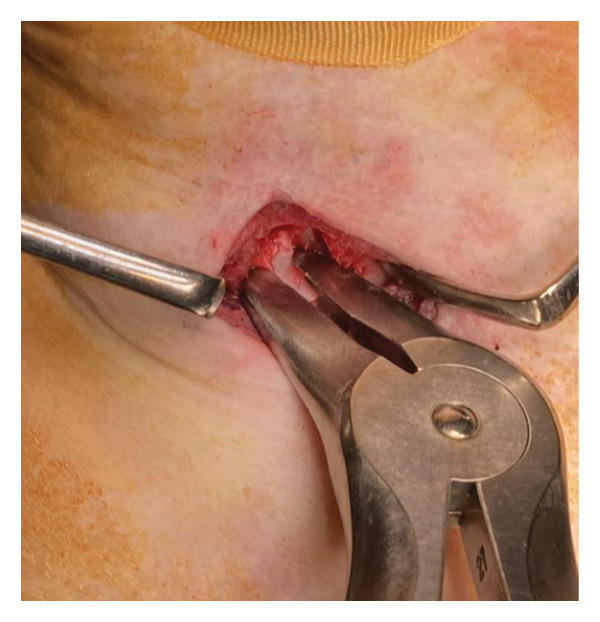
(c)

**Figure:**
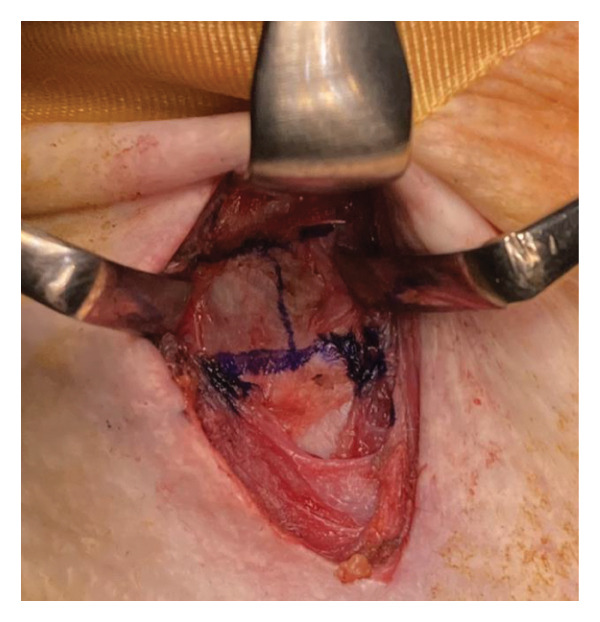
(d)

**Figure:**
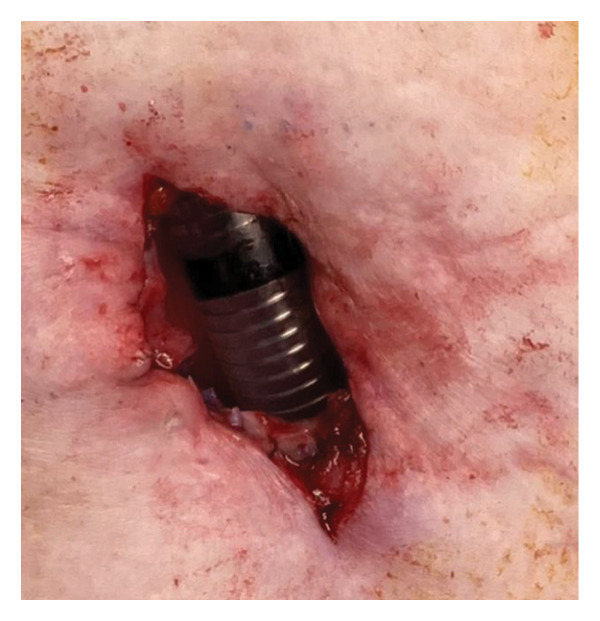
(e)

**Figure:**
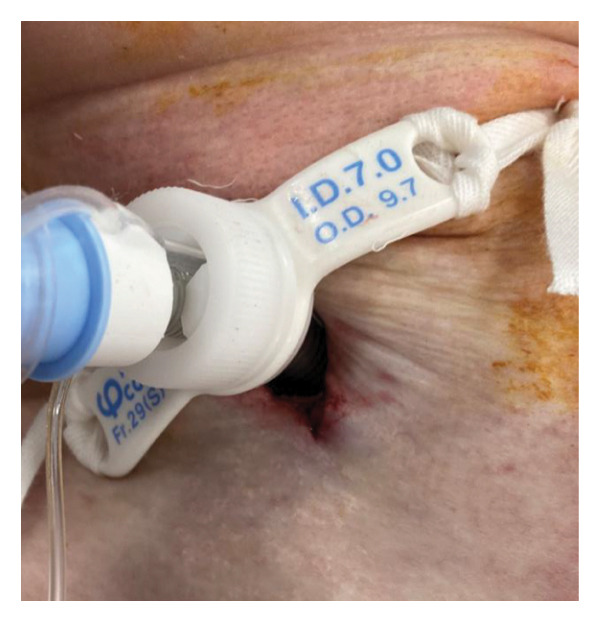
(f)

Tracheal access was achieved cranial to the endotracheal tube cuff, and no intraoperative cuff injury occurred. The tracheal mucosa was incised in a bipedicled, “book‐opening” fashion, followed by caudal extension of the tracheal cartilage incision, creating a stable stoma (Figure 2(d)). The cranial and caudal margins of the tracheal mucosa and resected cartilage were directly sutured to the skin using absorbable sutures (Figure 2(e)). A 7.0‐mm adjustable‐length, spiral‐reinforced silicone tracheostomy tube (Adjust Fit, Fuji Systems Corporation, Tokyo, Japan) was inserted without complications (Figure 2(f)).

As demonstrated in the postoperative chest radiograph, the tracheostomy tube was appropriately positioned at a high level (Figure 1(e)). Postoperatively, the airway remained stable, with no granulation tissue formation or subglottic stenosis observed. During a 6‐month follow‐up period, the tracheostoma was well‐formed without signs of infection or airway narrowing.

### 2.2. Case 2

A 60‐year‐old with achondroplasia complicated by profoundly severe scoliosis presented with significant anatomical challenges during airway management (Figures 3(a) and 3(b)). He was 117‐cm tall and weighed 44 kg, with a medical history of myasthenia gravis, Type 2 diabetes mellitus, and obstructive sleep apnea syndrome. After undergoing thymectomy for a thymoma, the patient developed worsening myasthenic symptoms and Type II respiratory failure, eventually requiring intubation. Due to the patient’s short neck and the caudal location of the trachea relative to the clavicle, the distance between the cricoid cartilage and the sternum was relatively short, measuring approximately 7 mm on CT images (Figure 3(c)). In addition, because adequate neck extension could not be achieved, surgical exposure was extremely limited. Based on these anatomical features, a high‐level tracheostomy was anticipated to be unavoidable in this case. If conventional tracheostomy had been performed between the cricoid cartilage and the first tracheal ring while preserving the cricoid cartilage, there would have been concern about postoperative tracheal stenosis due to granulation tissue formation. Therefore, cricotracheostomy was selected.

Figure 3Physical findings and radiological examinations in Case 2. (a) Photograph showing severe thoracic deformity and a short neck. (b) Computed tomography image demonstrating marked thoracic skeletal deformity. (c) Preoperative CT image showing the caudal position of the trachea relative to the clavicle and the short distance between the cricoid cartilage and the clavicle (approximately 7 mm). (d) Postoperative CT image showing appropriate placement of the tracheostomy tube at the level of the cricoid cartilage.

**Figure:**
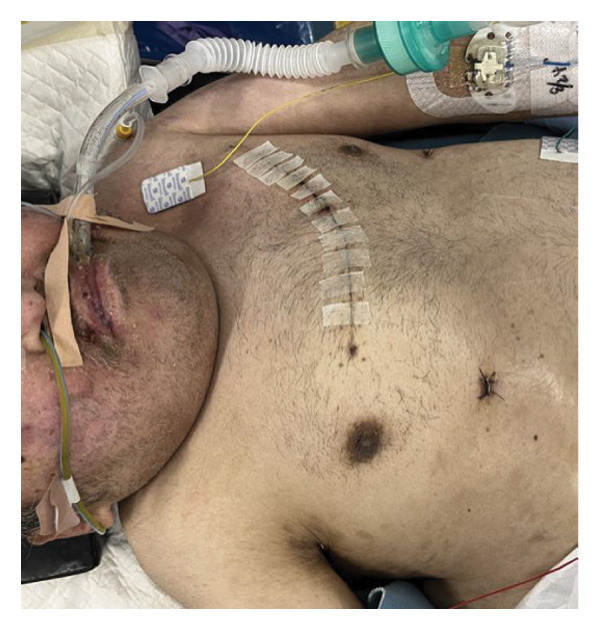
(a)

**Figure:**
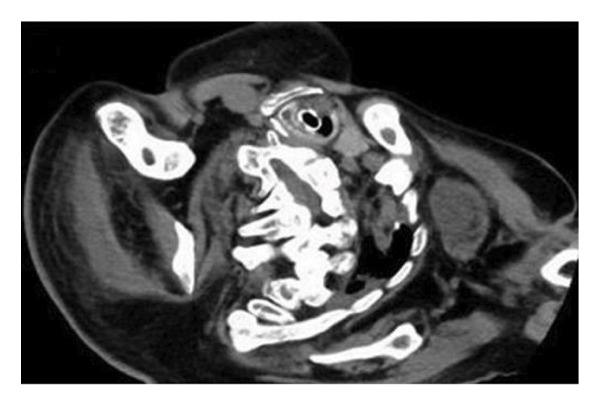
(b)

**Figure:**
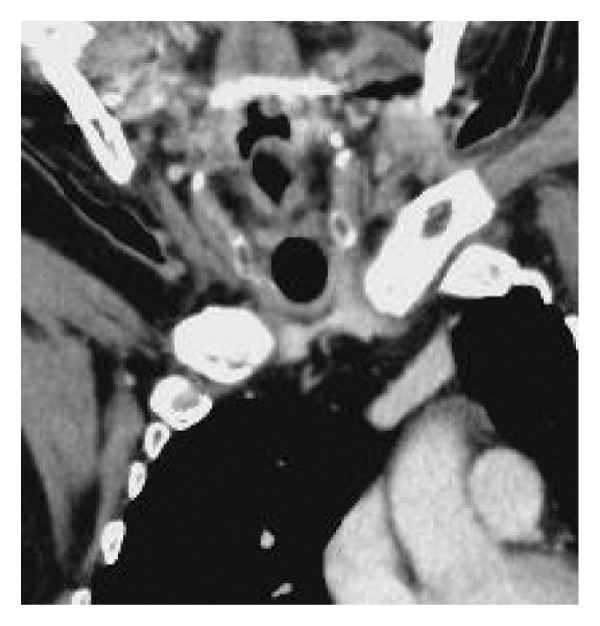
(c)

**Figure:**
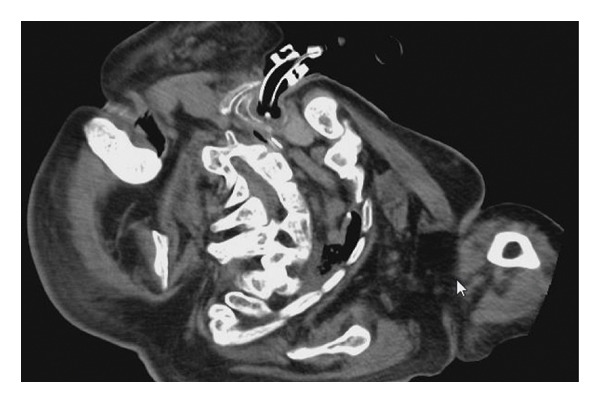
(d)

The patient was positioned using supportive cushions and secured with fixation bands on a tilted operating table to achieve maximal stability. A vertical midline incision was made directly over the cricoid cartilage, enabling its identification and partial resection of the anterior cricoid. Although the patient had a small body habitus and limited neck extension due to achondroplasia‐related skeletal deformity, no abnormalities in the anatomical relationships or vascular course were observed intraoperatively. No intraoperative cuff injury occurred. A stoma was created by suturing the resected cartilage and tracheal mucosa to the skin, with the cranial, caudal, and lateral margins directly secured using absorbable sutures. Subsequently, the adjustable‐length tracheostomy tube (Adjust Fit) was successfully inserted. Postoperative CT image shows appropriate placement of the tracheostomy tube at the level of the cricoid cartilage (Figure 3(d)). Mechanical ventilation was required after operation, and a cuffed tracheostomy tube was used. Mild tracheal mucosal erosion was observed; however, no progression was noted with monthly tube replacement.

## 3. Discussion

Cricotracheostomy avoids certain complications of conventional tracheostomy by partially resecting the anterior cricoid cartilage and securing the stoma at the level of the cricothyroid membrane [1]. Because the cricoid cartilage is typically well‐palpable and free of major anterior vascular structures, it serves as a reliable anatomical landmark even in patients with distorted cervical anatomy Tatekawa et al. reported the use of cricotracheostomy as a salvage procedure in a pediatric patient with congenital kyphoscoliosis since the tracheostomy tube was unstable during the conventional approach [3]. This demonstrates that partial cricoid resection can provide superior stoma stability in anatomically difficult cases. Accordingly, we adopted cricotracheostomy as the initial approach, rather than a rescue procedure, as a proactive measure to prevent such complications.

The two patients in this report had congenital severe scoliosis and skeletal deformities, making it particularly challenging to achieve airway access. Notably, the scoliosis was both anteroposterior and rotational, further limiting surgical exposure and neck positioning. One patient even had a markedly short neck, compounding the difficulty. Nevertheless, the cricoid cartilage was a consistent and palpable landmark in both cases, enabling safe and effective surgical access.

In addition, the Adjust Fit tracheostomy tube was used in both cases. This tube allows individualized length adjustment within the trachea using a movable adjuster, and its high flexibility and spiral reinforcement enable it to conform smoothly to the curved and deviated trachea in patients with severe skeletal deformities. The use of this tube may, therefore, reduce mechanical stress on the airway and facilitate safer long‐term airway management in such complex anatomical conditions.

Cricotracheostomy has the benefit of enabling tracheal access at a higher level, wherein the endotracheal cuff is less likely to be damaged during the procedure. This minimizes the risk of cuff rupture, enabling continuous positive pressure ventilation throughout surgery, which is a critical advantage in patients with respiratory failure. This higher access also mitigates the risk of developing a tracheoinnominate artery fistula after conventional tracheostomy, which is seen due to the tracheal deviation in severe scoliosis [6]. These benefits can also reduce surgeon anxiety and procedural uncertainty in such difficult cases. However, this technique involves resection of more than one third of the cricoid cartilage and represents a more invasive procedure than conventional tracheostomy. Therefore, in cases of temporary upper airway obstruction caused by conditions such as infection or allergy, conventional tracheostomy should remain the first‐line option. The indication for cricotracheostomy must be carefully evaluated.

Traditionally, high tracheostomy has been avoided due to the risk of cricoid cartilage compression. Nevertheless, by resecting the cricoid cartilage and suturing the tracheal stoma to the skin to create a well‐formed opening, granulation tissue formation may be reduced compared with conventional tracheostomy [5]. However, as a potential late complication, there remains a risk of subglottic stenosis, and regular follow‐up is required. In our cases, there were also no complications such as stenosis or infection during the relatively long‐term follow‐up, further supporting the durability of this technique.

Although the current evidence remains limited to case reports and small series, cricotracheostomy shows potential as an alternative to conventional tracheostomy in patients with chest wall and airway deformities.

## Consent

All the patients allowed personal data processing, and informed consent was obtained from all individual participants included in the study.

## Conflicts of Interest

The authors declare no conflicts of interest.

## Funding

This research did not receive any specific grant from funding agencies in the public, commercial, or not‐for‐profit sectors.

## Data Availability

The data that support the findings of this study are not publicly available due to privacy or ethical restrictions.
